# Transcriptomic profiling of burn patients reveals key lactylation-related genes and their molecular mechanisms

**DOI:** 10.3389/fmed.2025.1554791

**Published:** 2025-06-27

**Authors:** Yang Li, Jizhong Ma, Yeping Wang, Weibin Zhan, Qian Wang

**Affiliations:** Department of Burns and Plastic Surgery, Affiliated Jinhua Hospital, Zhejiang University School of Medicine, Jinhua, China

**Keywords:** lactylation, machine learning, burn, RPL14, SET, ENO1, PPP1CC

## Abstract

**Introduction:**

Burn injury is a global health concern characterized by complex pathophysiological changes. Understanding gene expression changes and molecular pathways, especially those related to lactylation, is crucial for developing effective treatments. This study aimed to analyze the transcriptomic profiles of burn patients and identify lactylation-related genes as potential biomarkers or therapeutic targets.

**Methods:**

Peripheral blood transcriptome data of burn patients and controls were obtained from the GEO database. After preprocessing to remove batch effects and normalize the data, differential genes were screened. Functional enrichment, lactylation gene analysis, machine learning for key gene selection, immune cell infiltration analysis, gene correlation and GSEA analysis, patient clustering, and upstream regulatory factor prediction were performed using various R packages. Statistical analysis was conducted using R software, with a *p*-value of < 0.05 considered significant.

**Results:**

Pathway enrichment analysis in burn patients showed significant alterations in immune-related pathways. Lactylation genes were differentially expressed, with changes in RNA processing and cell interactions. Machine learning identified four key lactylation-related molecules (RPL14, SET, ENO1, and PPP1CC). Immune microenvironment analysis revealed correlations with immune cell infiltration. Clustering analysis based on these four molecules divided burn patients into two subgroups, each exhibiting distinct gene expression patterns and pathway enrichments.

**Conclusion:**

This study provides insights into the molecular alterations in burn patients, especially regarding lactylation. The identified key molecules and pathways offer potential targets for personalized treatment. Future research should validate these findings and explore their clinical applications for improving burn patient management and prognosis.

## Introduction

Burn injury is a significant global health issue that often leads to complex pathophysiological changes in the body (1). The immune system and various molecular regulatory mechanisms play crucial roles in the body’s response to burn trauma (2–4). Understanding the alterations in gene expression (4) and related molecular pathways (5) in burn patients is essential for developing effective treatment strategies and improving patient outcomes.

Previous studies have demonstrated that burn injuries can initiate a cascade of inflammatory responses (6, 7), disrupting normal physiological processes (8). However, the detailed molecular mechanisms driving these changes, particularly those involving lactylation (9) and its associated regulatory networks, remain poorly understood. Lactylation (10, 11), a novel post-translational modification, has been implicated in a variety of biological processes (12), and its dysregulation may play a critical role in the pathophysiology of burn injuries.

In this study, we aimed to comprehensively analyze the transcriptomic profiles of peripheral blood samples from burn patients and normal controls. By integrating bioinformatics tools and machine learning algorithms, we sought to identify differentially expressed genes, with a particular focus on lactylation-related genes. We hypothesized that these genes could serve as potential biomarkers or therapeutic targets, providing valuable insights into the molecular mechanisms of burn injury and guiding the development of personalized treatment approaches.

## Methods

### Data source and preprocessing

Peripheral blood transcriptome data from burn patients (burn) and normal controls (control) were obtained from the public GEO database, including datasets GSE19743 (114 burn cases and 63 control cases) and GSE37069 (553 burn cases and 37 control cases). After merging, 767 samples of 18,884 genes were obtained.

R software (version 4.1.0) was used to analyze the data. Batch effects were removed using the R packages “limma (version 3.62.2)” and “sva (version 2.0.5),” and data normalization was performed using the “preprocessCore (version 1.68.0)” package in R to ensure data quality and comparability.

### Screening of differential genes

Differential expression analysis was conducted using the R package “limma (version 3.62.2),” with differentially expressed genes screened based on the criteria of adjusted *p*-value < 0.05 and|logFC| > 0.5.

### Functional enrichment analysis

For the differential genes, Gene Ontology (GO) annotation (including biological process, cellular component, and molecular function) and Kyoto Encyclopedia of Genes and Genomes (KEGG) pathway enrichment analysis were performed using the R package “clusterProfiler (version 4.14.4)” (13) to reveal gene functions and changes in related biological pathways.

### Analysis of lactylation genes

Lactylation modification gene sets were obtained from the literature (14, 15) (Supplementary Table S1), and the intersection with the differential genes was taken to obtain 25 upregulated lactylation genes and 55 downregulated lactylation genes. The GO and KEGG pathway enrichment analyses were then performed on these genes.

### Screening of key genes by machine learning

Three machine learning algorithms, XGBoost (XGBoost version 1.7.10.1) (16), random forest (RandomForest version 4.7–1.2) (17), and LASSO regression (glmnet version 4.1–8) (18), were used to analyze the differentially expressed genes. The correlation and predictive ability of these genes for disease occurrence were analyzed.

### Controlling overfitting in XGBoost and random Forest

To mitigate overfitting, distinct strategies were used in XGBoost and random forest models. For XGBoost, regularization was achieved through parameter tuning: max_depth = 6 was set to restrict tree complexity, while a learning rate (eta = 0.5) was applied to downweight individual tree contributions, implementing a gradient boosting shrinkage strategy. Additionally, default parameters, including gamma (minimum loss reduction required for splitting), subsample (random sampling of observations), and colsample_bytree (random sampling of features per tree), were leveraged to indirectly control overfitting.

For random forest, overfitting was addressed via three mechanisms: (1) Bootstrap sampling, where both observations and features were randomly sampled (with mtry = sqrt(p) specifying the default number of features per split) to enhance model diversity; (2) Optimal tree number selection, in which the code identified the minimum out-of-bag (OOB) error using which.min(rf$err.rate[,1]) to determine the optimal number of trees (optionTrees) and avoid redundancy; and (3) Feature randomness, whereby each tree utilized only a subset of features (via the default mtry parameter) to reduce inter-feature correlation and improve generalization. These approaches collectively ensured robust model performance while minimizing overfitting across both algorithms.

### Immune cell infiltration analysis

The ssGSEA function of the R package “GSVA (version 2.0.5)” (19) was used to evaluate the degree of immune cell infiltration, analyze the correlation of immune cell infiltration proportions, the differences between the patient and control groups, and the correlation between the four core genes and immune cell infiltration (only immune cells with a *p*-value of < 0.05 were shown).

### Gene correlation and GSEA analysis

Correlation analysis between the four core genes and all genes was performed, and the expression of the top 50 positively correlated genes was presented as a heatmap. Based on the results of the correlation analysis, GSEA analysis of the Reactome top 20 results of the four single genes was carried out using the R package “clusterProfiler (version 4.14.4),” and the enrichment score was calculated to determine the correlation between genes and pathways.

### Patient clustering and pathway analysis

Based on the four genes, unsupervised clustering typing of burn patients was performed using the R package “ConsensusClusterPlus (version 1.56.0),” and the optimal number of types was determined to be 2. The distribution of the two types of patients was shown by PCA plot, and the differential expression of the four genes between the different types was analyzed. The R package “pheatmap (version 1.0.12)” was used to draw a heatmap to show the relationship between clinical characteristics, gene expression, and typing. KEGG and Reactome pathways were downloaded from the Msigdb database (20), and pathway scoring was performed using the R package “GSVA (version 2.0.5).” The differences in pathways between the two types were compared, and heatmaps were drawn to show the comparison.

### Prediction of upstream regulatory factors

The regnetwork database^1^ was used to predict miRNAs and transcription factors upstream of the genes, and the R packages “igraph (version 2.1.4) + ggraph (version 2.2.1)” were used to construct the regulatory network.

### Statistical analysis

R software (Version 4.1.0) was used for graphing, calculations, and statistical analysis of the results. Student’s t-test was utilized to compare the mean values of different groups, and we used the Benjamini–Hochberg false discovery rate to correct multiple comparisons. To compare immune cell infiltration levels between groups, the Wilcoxon rank-sum test was used. For pathway enrichment analysis across clusters, Fisher’s exact test was used. Statistical significance was set at a *p*-value of <0.05.

## Results

### Abnormal enrichment of pathways in burn patients

**Figure 1:**
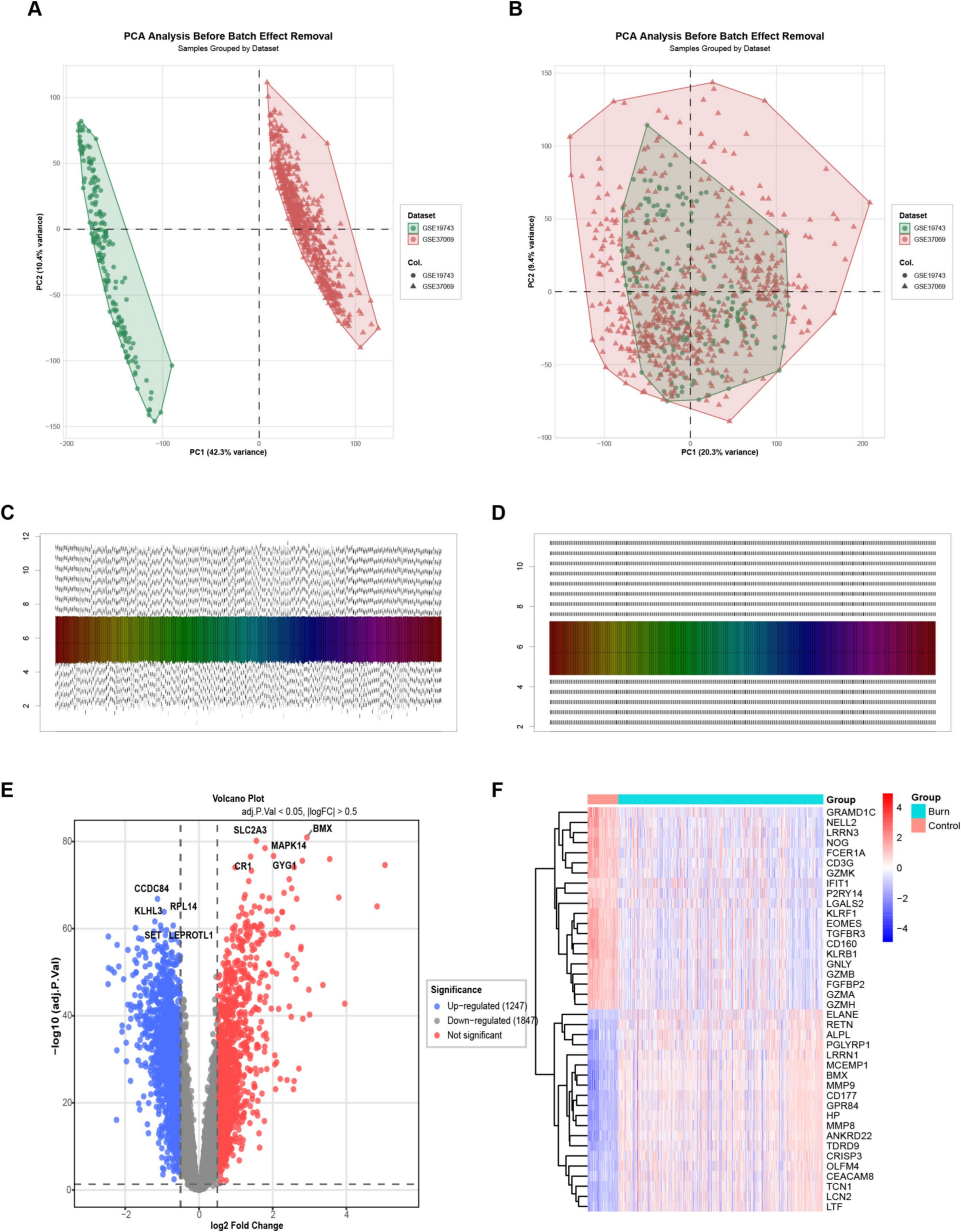
Batch effect removal and data normalization. **(A)** Scatter plot of gene expression data before batch effect removal, showing a relatively scattered distribution; **(B)** Scatter plot of gene expression data after batch effect, with data points more clustered and a more reasonable distribution; **(C)** Box plot of gene expression data before normalization, indicating inconsistent data scales; **(D)** Box plot of gene expression data after normalization, with unified data scales; **(E)** Volcano plot of differential gene analysis. Blue dots represent downregulated genes, and red dots represent upregulated genes, screened based on *p*-value < 0.05 and |logFC| > 0.5; **(F)** Heatmap shows the differences in gene expression between healthy and burn groups. Red indicates higher expression, and blue indicates lower expression, demonstrating the significant differences in gene expression between the two groups.

To compare the differences in lactylation-related genes between burn patients and normal patients, we analyzed the peripheral blood transcriptomics of these two types of patients. For the 18,884 genes from 767 samples after combining two datasets, we first removed the batch effect. Before removal (Figure 1A), the data distribution was rather scattered, while after removal (Figure 1B), the data points were more clustered and the distribution was more reasonable, effectively reducing batch-related errors. Subsequently, data normalization was carried out. Before normalization (Figure 1C), the data scales were inconsistent, and after normalization (Figure 1D), the data scales were unified, facilitating the accurate comparison of gene expression levels in the subsequent steps. Finally, differential genes were screened according to the criteria of adjusted *p*-value < 0.05 and|logFC| > 0.5. As a result, 1,247 upregulated genes and 1,847 downregulated genes were obtained. The volcano plot (Figure 1E) showed the distribution of differential genes, and the heatmap (Figure 1F) demonstrated the differences in gene expression between the healthy and burn groups, indicating that there were significant differences in gene expression between the two groups.

**Figure 2:**
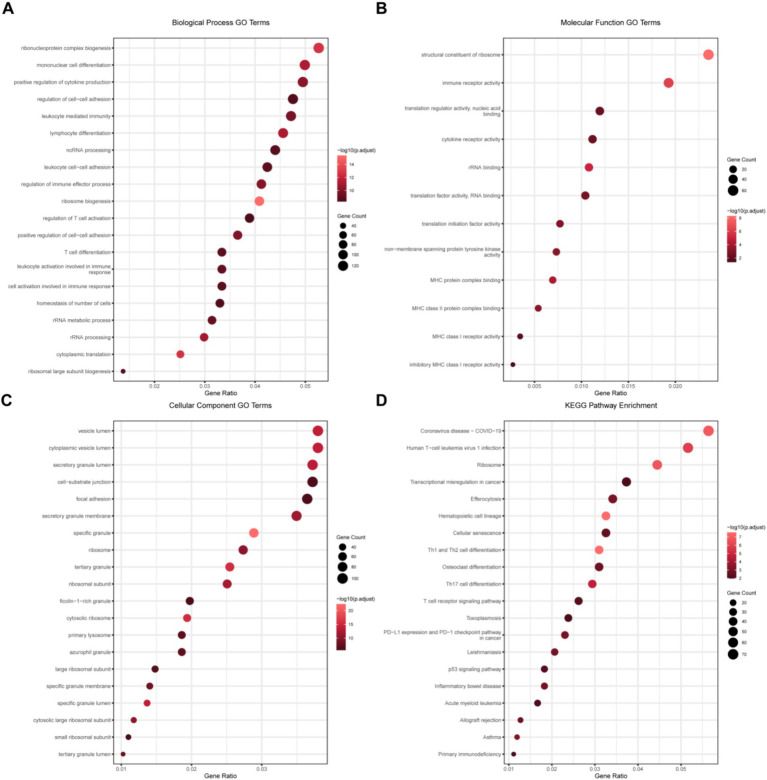
GO and KEGG enrichment analyses of differential genes. **(A)** GO analysis of the biological process (BP) of differential genes; **(B)** GO analysis of the cellular component (CC) of differential genes; **(C)** GO analysis of the molecular function (MF) of differential genes; and **(D)** KEGG pathway enrichment analysis results of the intersection of differential genes.

Subsequently, we performed the GO/KEGG enrichment analyses on the differential genes. The GO analysis of biological processes (Figure 2A) enriched inflammation-related pathways such as “mononuclear cell differentiation,” “positive regulation of cytokine production,” and “leukocyte mediated immunity,” which proved that the immune statuses of the two groups of patients were significantly different. Similarly, in the GO analysis of molecular functions (Figure 2B), immune-related changes were also enriched. In the GO analysis of cellular components (Figure 2C), structural changes in many transport-related organelles were enriched, which was in line with the pathological changes in burn patients. The KEGG analysis (Figure 2D) showed that helper T cells had relatively large changes, suggesting that helper T cells play an important role in the pathological process of burns.

### Changes in Lactylation genes in burn patients

**Figure 3:**
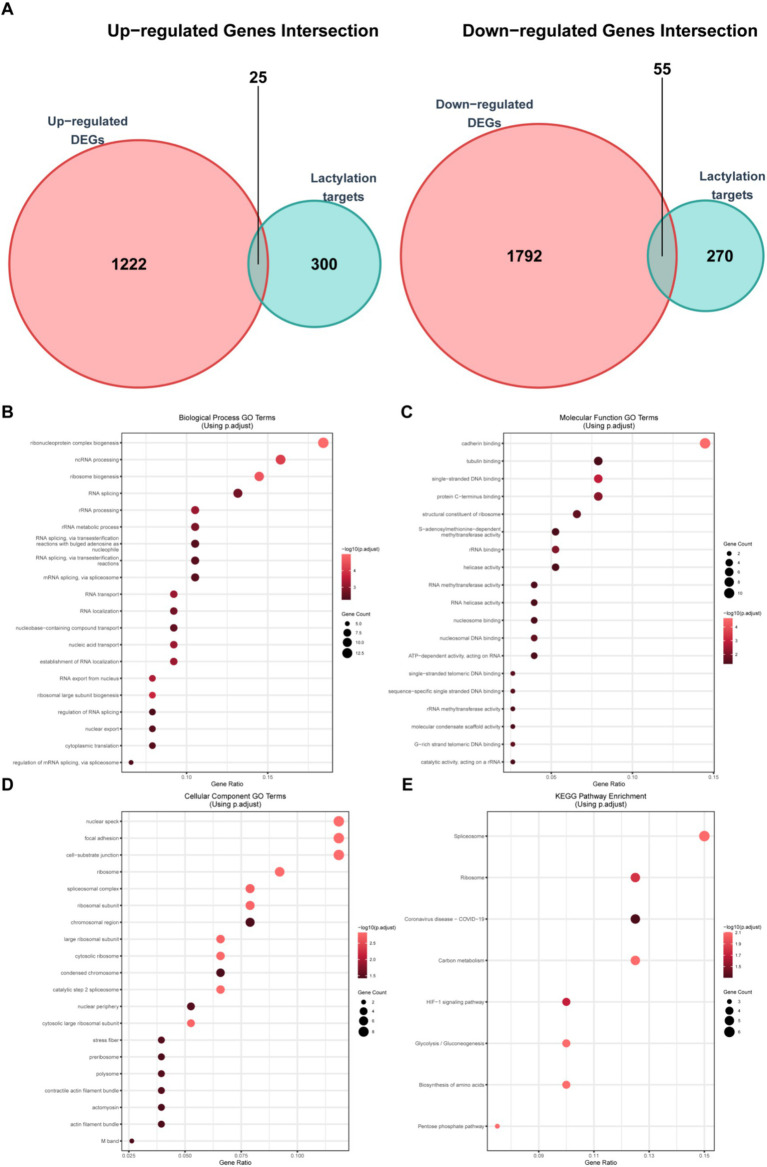
Analysis of lactylation-related differential genes. **(A)** Venn diagram shows the intersection of the upregulated gene set and the lactylation gene set (25 genes) and the intersection of the downregulated gene set and the lactylation gene set (55 genes); **(B)** GO analysis of the biological process (BP) of lactylation-related differential genes; **(C)** GO analysis of the cellular component (CC) of lactylation-related differential genes; **(D)** GO analysis of the molecular function (MF) of lactylation-related differential genes; and **(E)** KEGG pathway enrichment analysis results of lactylation-related differential genes.

Since lactylation plays an important role in biological processes, we took the intersection of the upregulated (Figure 3A) and downregulated (Figure 3B) differential genes with the lactylation gene set (Supplementary Table S1), obtaining 25 upregulated and 55 downregulated lactylation genes (Supplementary Figure S1A), which were then presented by heatmap (Supplementary Figure S1B) and box plot (Supplementary Figure S1C). Subsequently, we performed the GO/KEGG analyses on these differential genes. The GO analysis of biological processes (Figure 3C) revealed enrichment in RNA change-related pathways such as “ncRNA processing,” “RNA splicing,” “rRNA processing,” and “rRNA metabolic process,” demonstrating significant alterations in RNA metabolism in burn patients. Interestingly, in the GO analysis of molecular functions (Figure 3D), functions related to binding were significantly enriched, indicating changes in cell interactions. GO analysis of cellular components (Figure 3E) further proved that RNA splicing and related structures had changed. KEGG analysis (Figure 3E) showed that metabolic and HIF-1 signaling pathways had changed. The above data indicated that the lactylation-related genes in burn patients had significant changes compared to controls, potentially impacting RNA processing and other critical cellular functions.

### Screening of key Lactylation-related molecules in burn patients by machine learning

**Figure 4:**
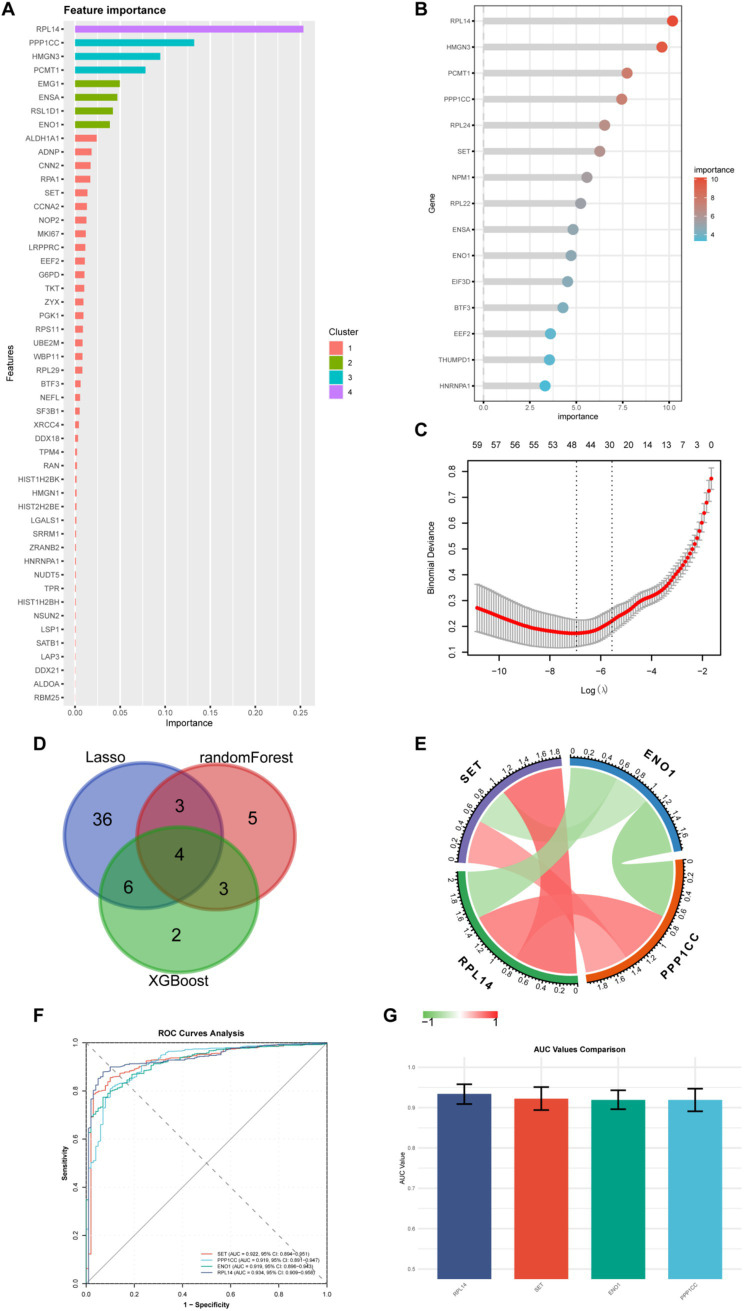
Screening of key genes by machine learning. **(A)** Importance ranking of 15 genes selected by the XGBoost algorithm; **(B)** Importance ranking of 15 genes selected by the random forest algorithm; **(C)** 49 genes screened using LASSO regression; **(D)** Venn diagram shows the intersection of genes selected by the three machine learning algorithms, obtaining four core genes; **(E)** Correlation heatmap of the four core genes. Red represents positive correlation, and green represents negative correlation; **(F)** ROC curves of the four genes for predicting disease occurrence; The X-axis is “1-specificity,” and the Y-axis is “sensitivity.” Different genes have different sensitivities and specificities, and the area under the ROC curve (AUC) reflects the predictive ability of the gene for disease occurrence; **(G)** AUC values of the ROC curves of the four genes.

To identify the key molecules, we adopted machine learning, including XGBoost, Random Forest, and LASSO regression. XGBoost was chosen for its efficiency in handling large-scale datasets and ability to capture non-linear relationships, making it suitable for complex gene-disease association analysis (21). Random Forest was selected due to its robustness against overfitting and interpretability via feature importance scores, which aids in identifying critical genes with high predictive power for disease occurrence (17). LASSO regression was utilized to perform dimensionality reduction and feature selection by imposing a penalty on model complexity, effectively narrowing down genes through sparse regularization (22). The XGBoost (Figure 4A), random forest (Figure 4B), and LASSO regression (Figure 4C) models were used to select 15, 15, and 49 lactylation-related genes, respectively. In both the XGBoost and random forest models, the gene with the highest weight selected was RPL14. Subsequently, we took the intersection of the molecules selected by the three machine learning models (Figure 4D) and obtained four common key molecules, namely RPL14, SET, ENO1, and PPP1CC. Through correlation analysis (Figure 4E), we found that ENO1 was negatively correlated with the other three genes, while the other three genes were positively correlated with each other. Subsequently, we used these four molecules to distinguish burn patients (Figure 4F). The AUC values from high to low were as follows: RPL14 (AUC = 0.934), SET (AUC = 0.922), ENO1 (AUC = 0.919), and PPP1CC (AUC = 0.919) (Figure 4G). In addition, we also compared the above four indicators with the well-recognized burn-related indicators IL-6, TNF-*α*, and CRP (Supplementary Figure S2). The results showed that the above AUC values were all greater than 0.9, indicating that these four molecules have a better ability to distinguish burn patients.

### Regulation of the immune microenvironment and different pathways by key Lactylation-related molecules

**Figure 5:**
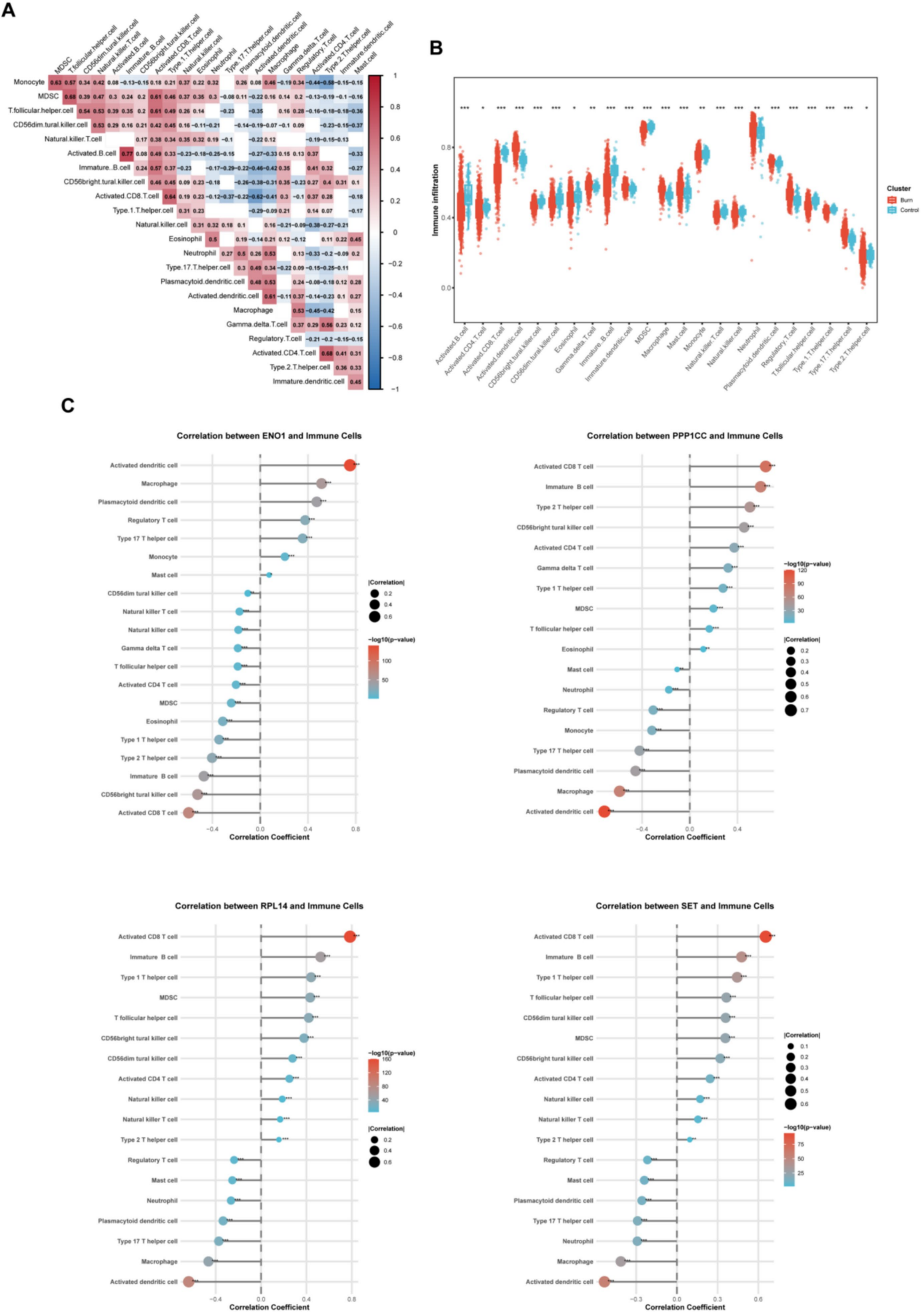
Evaluation of immune cell infiltration. **(A)** Heatmap shows the correlation of immune cell infiltration proportions; **(B)** Box plot shows the differences in immune cell infiltration between the patient and control groups; **(C)** Scatter plot shows the correlation between the four core genes and immune cell infiltration. The size of the circle represents the correlation coefficient, and the color represents the *p*-value.

Next, by exploring changes in the immune microenvironment, we found that activated CD8^+^T cells were most closely correlated with myeloid-derived suppressor cells (MDSCs) and follicular helper T cells (Figure 5A). Comparing burn patients with healthy individuals (Figure 5B), we found that the number of activated DC (dendritic cell) cells in burn patients significantly increased, while the numbers of activated CD8^+^T cells, B cells, and CD4^+^T cells were all decreased. In addition, the numbers of neutrophils and Treg cells in burn patients were significantly increased, reflecting the body’s efforts to maintain the balance of the immune system. Subsequently, we analyzed the relationship between the four key molecules and the immune microenvironment (Figure 5C). The cells that were most significantly positively and negatively correlated with ENO1 were activated DC cells and activated CD8^+^T cells, respectively. Interestingly, the other three molecules exhibited the opposite pattern, further confirming the negative correlation between ENO1 and the other three molecules.

**Figure 6:**
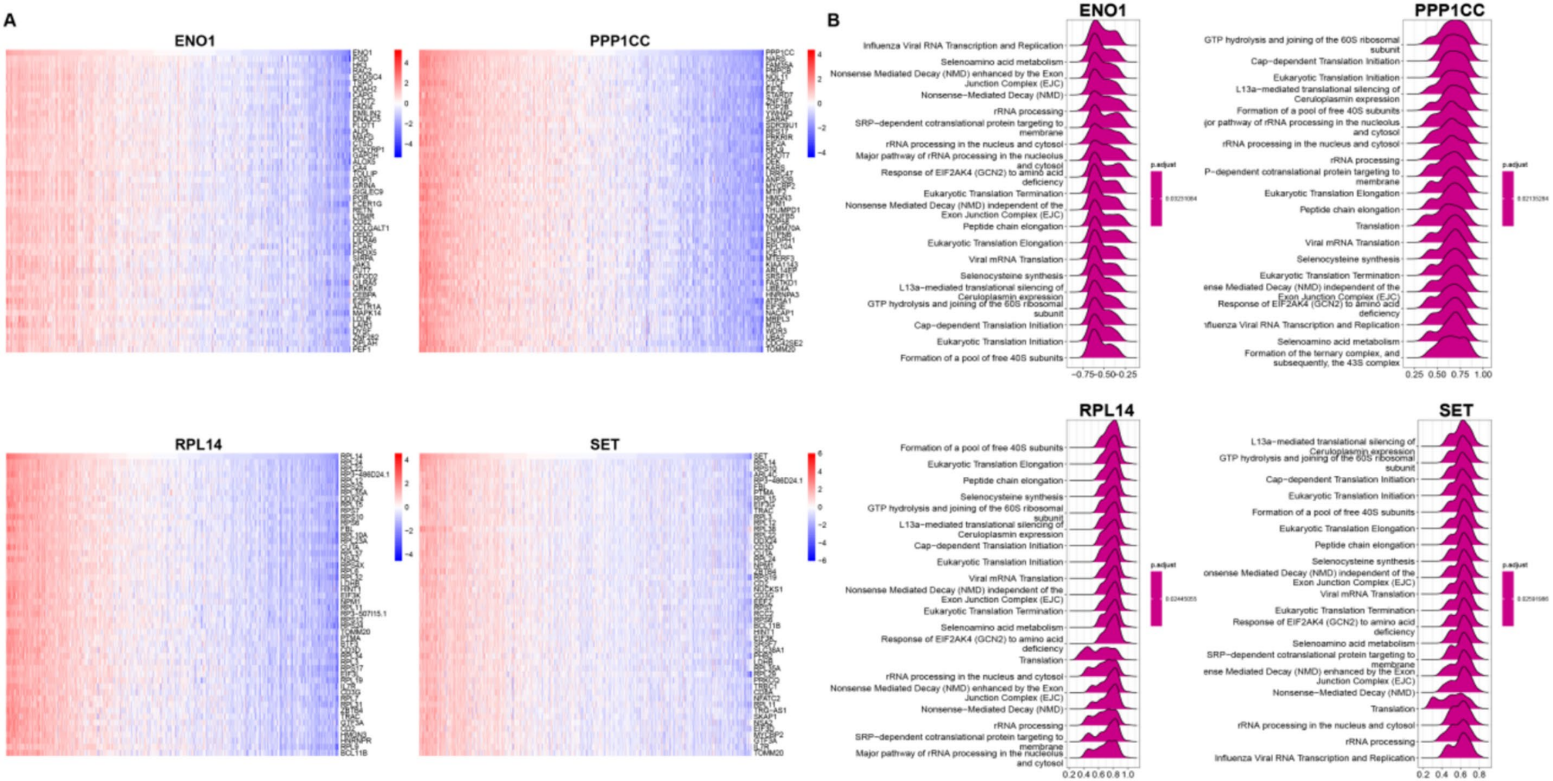
Characteristics of lactylation-related genes. **(A)** Correlation Analysis between Four Genes and All Genes Heatmap shows the expression of the top 50 positively correlated genes in the correlation analysis between the four genes and all genes; **(B)** GSEA Analysis of Four Single GenesReactome top 20 GSEA analysis results of the four single genes based on the correlation analysis in top 50 positively correlated genes. The values below represent the enrichment score. A positive score indicates a positive correlation between the gene and the pathway, and a negative score indicates a negative correlation.

We performed correlation analyses between ENO1, PPP1CC, RPL14, SET, and other genes, selecting the top 50 for display (Figure 6A). Based on these correlations, we conducted single-gene GSEA analysis using Reactome (Figure 6B). Interestingly, although ENO1 was negatively correlated with the other three molecules, all four molecules were enriched in the “rRNA processing” pathway, indicating significant changes in the ribosome function in burn patients. Moreover, we analyzed the gene regulatory network of these four molecules (Supplementary Figure S3), from which the complexity of the regulatory network and the regulation by has-miRNA could be observed.

### Identification of Lactylation-related molecular subgroups and differences between subgroups

**Figure 7:**
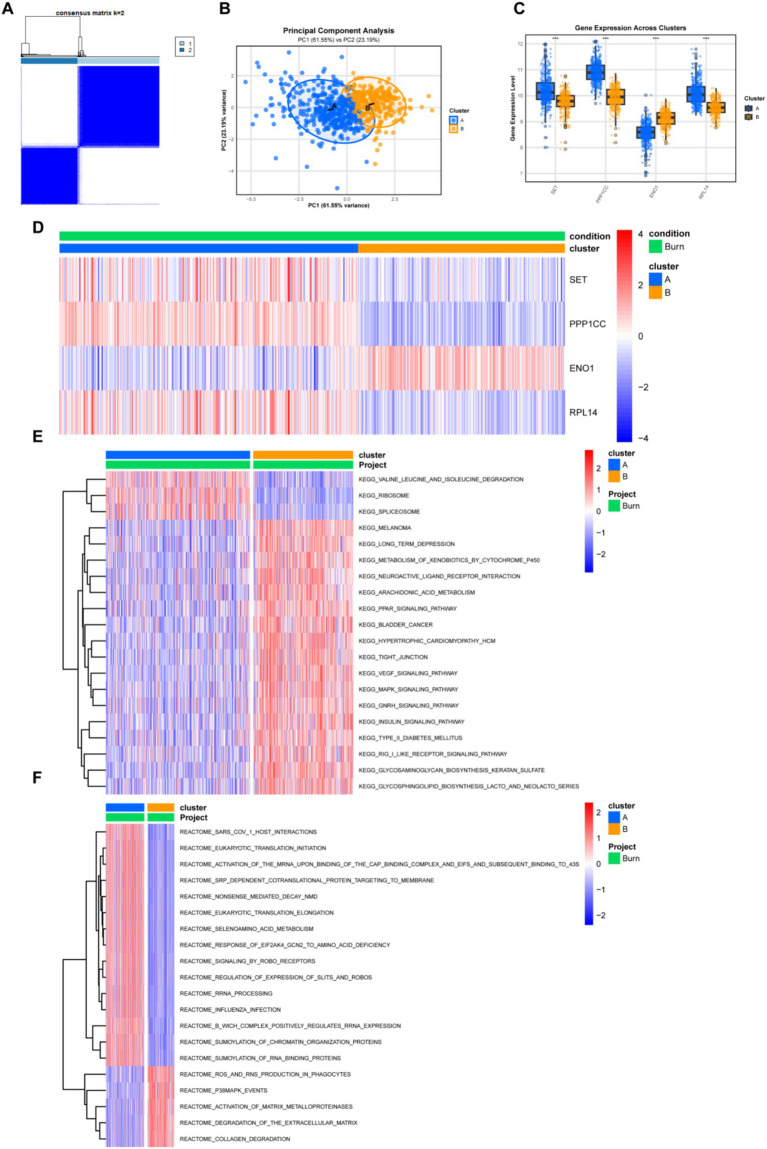
Clustering and pathway analysis of burn patients. **(A)** Unsupervised clustering typing of burn patients based on four genes, with the optimal number of types being 2; **(B)** PCA plot shows the distribution of the two types of patients; **(C)** Box plot shows the differential expression of the four genes between different types; **(D)** Heatmap shows the relationship between clinical characteristics, gene expression, and typing; **(E,F)** Heatmaps comparing the differences in the KEGG and Reactome pathways between the two types of patients. Pathway scoring was performed after downloading the pathways from the Msigdb database.

To explore the relationship between the expression of the four lactylation-related molecules and the subgroups of burn patients, we performed a consensus clustering analysis on all burn patients. By increasing the clustering variable (*k*) from 2 to 10, we found that when *k* = 2, the within-group correlation was the highest and the between-group correlation was relatively low, indicating that burn patients could be well divided into two clusters based on the above four molecules (Figure 7A). PCA analysis showed that patients in Cluster A and Cluster B could be distinguished by this clustering method (Figure 7B). Subsequently, we explored the inter-cluster expression patterns of the four genes and found that there were significant differences in the expression of SET, PPP1CC, ENO1, and RPL14 between the clusters. Cluster A exhibited higher expression levels of SET, PPP1CC, and RPL14, while Cluster B was characterized by enhanced expression of ENO1 (Figure 7C). The heatmap (Figure 7D) showed that there were slightly more patients in Cluster A than in Cluster B, and the expression patterns of the four molecules in the two groups of patients were completely different. The KEGG pathway analysis (Figure 7E) of the two groups of patients showed that pathways such as “VALINE_LEUCINE_AND_ISOLEUCINE_DEGRADATION,” “RIBOSOME,” and “SPLICEOSOME” were upregulated in Cluster A, while pathways such as “PPAR,” “VEGF,” and “MAPK” were enriched in Cluster B. The Reactome pathway analysis (Figure 7F) showed that RNA remodeling was very active in Cluster A, while extracellular matrix degradation and remodeling were active in Cluster B. The above data indicated that these four lactylation-related molecules could significantly distinguish different burn patients and might be beneficial for the stratified treatment of burn patients.

## Discussion

In this study, we conducted a comprehensive analysis of the peripheral blood transcriptomes of burn patients and normal controls. Through a series of bioinformatics and machine learning techniques, we identified significant differences in gene expression and pathway enrichment between the two groups, with a particular emphasis on lactylation-related genes (SET, PPP1CC, ENO1, and RPL14).

The observed changes in immune-related pathways (3, 23) and the immune microenvironment (24) in burn patients are consistent with the known pathophysiology of burn injuries (25, 26). The upregulation of inflammation-related pathways and the alterations in immune cell populations highlight the body’s attempt to mount an immune response and maintain homeostasis (27). The dysregulation of lactylation genes may further modulate these processes, potentially affecting the severity and outcome of burn injuries.

Lactylation, a newly discovered post-translational modification, is closely associated with cellular metabolic states, particularly lactate metabolism (28, 29). Burn injury typically triggers severe systemic inflammation and metabolic disorders, during which lactate accumulation may occur due to tissue hypoxia, glycolytic pathway activation, or immune cell dysfunction (30, 31). The dysregulation of lactylation-related genes observed in this study suggests that abnormal lactylation modification may participate in the molecular mechanisms underlying burn injury by affecting multiple biological processes. The identification of four key lactylation-related molecules (RPL14, SET, ENO1, and PPP1CC) using machine learning algorithms provides novel insights into the molecular mechanisms underlying burn injury. According to our analysis, these molecules not only showed strong predictive power for distinguishing burn patients but also exhibited complex regulatory relationships with each other and with the immune microenvironment (32, 33). Among them, ENO1, as a glycolytic enzyme, can play a tumor-promoting role in a variety of tumors (34–36), which may indicate that the type B burn patients identified in this article have a poorer prognosis. The negative correlation between ENO1 and the other three molecules, along with their shared enrichment in the rRNA processing pathway, suggests a coordinated regulatory network that may be crucial for cellular function and response to burn stress (37, 38).

Furthermore, the immune microenvironment analysis in this study revealed correlations between lactylation-related genes and immune cell infiltration. For instance, ENO1, a key gene involved in glycolysis, was negatively correlated with activated CD8 + T cells but positively correlated with activated dendritic cells (39). This may indicate that lactylation modulates immune cell function by regulating metabolic pathways. In the context of burn injury, excessive lactate production may induce an immunosuppressive microenvironment through lactylation modification, thereby inhibiting T cell function while promoting the activation of myeloid-derived suppressor cells (MDSCs) or dendritic cells (30, 40), ultimately affecting the body’s anti-infection ability and wound healing.

The clustering analysis based on these four molecules revealed two distinct subgroups of burn patients, each with characteristic gene expression patterns and pathway enrichments (41, 42). This finding implies the potential for personalized treatment strategies tailored to the specific molecular profiles of individual patients. For example, patients in Cluster A, with upregulated VALINE_LEUCINE_AND_ISOLEUCINE_DEGRADATION, RIBOSOME, and SPLICEOSOME pathways, may respond differently to interventions compared to those in Cluster B, where pathways such as PPAR, VEGF, and MAPK are enriched. These data may provide favorable scientific evidence for the treatment of these patients with pathway inhibitors.

The predicted regulatory network involving miRNAs and transcription factors provides a broader context for understanding the transcriptional and post-transcriptional regulation of lactylation-related genes (43). This network may offer additional targets for therapeutic modulation (44) and further investigation into the complex regulatory mechanisms in burn injury.

However, our study has several limitations. The transcriptomic data were derived from peripheral blood samples, which may not fully represent the molecular changes occurring in the burn-injured tissues. Then, the clinical significance of the subgroups was not analyzed due to the absence of clinical data available in this study. Additionally, further experimental validation, such as functional studies and *in vivo* models, is required to confirm the biological significance of the identified genes and pathways. Longitudinal studies are also needed to monitor the dynamic changes in gene expression and the immune microenvironment over the course of burn injury and recovery.

## Conclusion

Our study provided valuable insights into the molecular alterations in burn patients, particularly those related to lactylation. The identified key molecules and pathways offer potential targets for future research and the development of personalized treatment strategies. Future studies should focus on validating these findings and exploring the clinical applications of these molecular signatures in improving the management and prognosis of burn patients.

## Data Availability

The original contributions presented in the study are included in the article/Supplementary material, further inquiries can be directed to the corresponding author.

## Footnotes

^1^ https://figshare.com/s/566ab5e5e340e988b6c6

## References

[1] Bailey ME Sagiraju HKR Mashreky SR Alamgir H. Epidemiology and outcomes of burn injuries at a tertiary burn care center in Bangladesh. Burns. (2019) 45:957–63. doi: 10.1016/j.burns.2018.12.011, PMID: 30612889

[2] Mahung C Wallet SM Jacobs JE Zhou LY Zhou H Cairns BA. Multiplexed human gene expression analysis reveals a central role of the TLR/mTOR/PPARγ and NFkB axes in burn and Inhalation injury-induced changes in systemic Immunometabolism and Long-term patient outcomes. Int J Mol Sci. (2022) 23:9418. doi: 10.3390/ijms23169418, PMID: 36012680 PMC9409318

[3] Wang P Zhang Z Yin B Li J Xialin C Lian W. Identifying changes in immune cells and constructing prognostic models using immune-related genes in post-burn immunosuppression. PeerJ. (2022) 10:e12680. doi: 10.7717/peerj.12680, PMID: 35070500 PMC8761370

[4] Mahung C Stepp WH Long C Malfitano M Saklayici I Wallet SM. Early expression of IL-10, IL-12, ARG1, and NOS2 genes in peripheral blood mononuclear cells synergistically correlate with patient outcome after burn injury. J Trauma Acute Care Surg. (2022) 93:702–11. doi: 10.1097/ta.0000000000003602, PMID: 35363228 PMC9522922

[5] Liu X Rong Y Huang D Liang Z Yi X Wu F. Altered genes and biological functions in response to severe burns. Biomed Res Int. (2021) 2021:8836243. doi: 10.1155/2021/8836243, PMID: 34124262 PMC8168476

[6] Lord JM Midwinter MJ Chen YF Belli A Brohi K Kovacs EJ. The systemic immune response to trauma: an overview of pathophysiology and treatment. Lancet. (2014) 384:1455–65. doi: 10.1016/s0140-6736(14)60687-5, PMID: 25390327 PMC4729362

[7] Jackson SP Darbousset R Schoenwaelder SM. Thromboinflammation: challenges of therapeutically targeting coagulation and other host defense mechanisms. Blood. (2019) 133:906–18. doi: 10.1182/blood-2018-11-882993, PMID: 30642917

[8] Abdullahi A Amini-Nik S Jeschke MG. Animal models in burn research. Cell Mol Life Sci. (2014) 71:3241–55. doi: 10.1007/s00018-014-1612-5, PMID: 24714880 PMC4134422

[9] Barayan D Abdullahi A Knuth CM Khalaf F Rehou S Screaton RA. Lactate shuttling drives the browning of white adipose tissue after burn. Am J Physiol Endocrinol Metab. (2023) 325:E180–e191. doi: 10.1152/ajpendo.00084.2023, PMID: 37406182 PMC10396278

[10] Li H Sun L Gao P Hu H. Lactylation in cancer: current understanding and challenges. Cancer Cell. (2024) 42:1803–7. doi: 10.1016/j.ccell.2024.09.006, PMID: 39393355

[11] Jiang X Yang Y Li X Li T Yu T Fu X. Lactylation: An innovative approach to disease control. Aging Dis. (2024). doi: 10.14336/ad.2024.0918, PMID: 39325940 PMC12221411

[12] Yu X Yang J Xu J Pan H Wang W Yu X. Histone lactylation: from tumor lactate metabolism to epigenetic regulation. Int J Biol Sci. (2024) 20:1833–54. doi: 10.7150/ijbs.91492, PMID: 38481814 PMC10929197

[13] Xu S Hu E Cai Y Xie Z Luo X Zhan L. Using clusterProfiler to characterize multiomics data. Nat Protoc. (2024) 19:3292–320. doi: 10.1038/s41596-024-01020-z, PMID: 39019974

[14] Cheng Z Huang H Li M Liang X Tan Y Chen Y. Lactylation-related gene signature effectively predicts prognosis and treatment responsiveness in hepatocellular carcinoma. Pharmaceuticals. (2023) 16:644. doi: 10.3390/ph16050644, PMID: 37242427 PMC10221268

[15] Wan N Wang N Yu S Zhang H Tang S Wang D. Cyclic immonium ion of lactyllysine reveals widespread lactylation in the human proteome. Nat Methods. (2022) 19:854–64. doi: 10.1038/s41592-022-01523-1, PMID: 35761067

[16] Dou B Zhu Z Merkurjev E Ke L Chen L Jiang J. Machine learning methods for small data challenges in molecular science. Chem Rev. (2023) 123:8736–80. doi: 10.1021/acs.chemrev.3c00189, PMID: 37384816 PMC10999174

[17] Hu J Szymczak S. A review on longitudinal data analysis with random forest. Brief Bioinform. (2023) 24:bbad002. doi: 10.1093/bib/bbad002, PMID: 36653905 PMC10025446

[18] Wang Y Zou B Xu J Xu C Tang YY. ALR-HT: a fast and efficient lasso regression without hyperparameter tuning. Neural Netw. (2025) 181:106885. doi: 10.1016/j.neunet.2024.106885, PMID: 39546876

[19] Hänzelmann S Castelo R Guinney J. GSVA: gene set variation analysis for microarray and RNA-seq data. BMC Bioinformatics. (2013) 14:7. doi: 10.1186/1471-2105-14-7, PMID: 23323831 PMC3618321

[20] Liberzon A Birger C Thorvaldsdóttir H Ghandi M Mesirov JP Tamayo P. The molecular signatures database (MSigDB) hallmark gene set collection. Cell Syst. (2015) 1:417–25. doi: 10.1016/j.cels.2015.12.004, PMID: 26771021 PMC4707969

[21] Liang D Wang L Zhong P Lin J Chen L Chen Q. Perspective: global burden of iodine deficiency: insights and projections to 2050 using XGBoost and SHAP. Adv Nutr. (2025) 16:100384. doi: 10.1016/j.advnut.2025.100384, PMID: 39914495 PMC11909719

[22] Lee S Seo MH Shin Y. The lasso for high dimensional regression with a possible change point. J R Stat Soc Series B Stat Methodol. (2016) 78:193–210. doi: 10.1111/rssb.12108, PMID: 27656104 PMC5014306

[23] Sierawska O Małkowska P Taskin C Hrynkiewicz R Mertowska P Grywalska E. Innate immune system response to burn damage-focus on cytokine alteration. Int J Mol Sci. (2022) 23:716. doi: 10.3390/ijms23020716, PMID: 35054900 PMC8775698

[24] Wang P Zhang Z Lin R Lin J Liu J Zhou X. Machine learning links different gene patterns of viral infection to immunosuppression and immune-related biomarkers in severe burns. Front Immunol. (2022) 13:1054407. doi: 10.3389/fimmu.2022.1054407, PMID: 36518755 PMC9742460

[25] Zhang F Qiu XC Wang JJ Hong XD Wang GY Xia ZF. Burn-related dysregulation of inflammation and immunity in experimental and clinical studies. J Burn Care Res. (2017) 38:e892–9. doi: 10.1097/bcr.0000000000000511, PMID: 28296672

[26] Seim RF Mac M Sjeklocha LM Kwiatkowski AJ Keselowsky BG Wallet SM. Nuclear Factor-Erythroid-2-Related factor regulates systemic and pulmonary barrier function and immune programming after burn and inhalation injury. Shock. (2023) 59:300–10. doi: 10.1097/shk.0000000000002022, PMID: 36730842 PMC9957943

[27] Moins-Teisserenc H Cordeiro DJ Audigier V Ressaire Q Benyamina M Lambert J. Severe altered immune status after burn injury is associated with bacterial infection and septic shock. Front Immunol. (2021) 12:586195. doi: 10.3389/fimmu.2021.586195, PMID: 33737924 PMC7960913

[28] Li X Yang Y Zhang B Lin X Fu X An Y. Lactate metabolism in human health and disease. Signal Transduct Target Ther. (2022) 7:305. doi: 10.1038/s41392-022-01151-3, PMID: 36050306 PMC9434547

[29] Zhang D Tang Z Huang H Zhou G Cui C Weng Y. Metabolic regulation of gene expression by histone lactylation. Nature. (2019) 574:575–80. doi: 10.1038/s41586-019-1678-1, PMID: 31645732 PMC6818755

[30] Chen AN Luo Y Yang YH Fu JT Geng XM Shi JP. Lactylation, a novel metabolic reprogramming code: current status and prospects. Front Immunol. (2021) 12:688910. doi: 10.3389/fimmu.2021.688910, PMID: 34177945 PMC8222712

[31] Efejuku TA Obanigba G Johnson D Obi A Hallman T Song J. Impact of pre-burn statin use on metabolic and cardiovascular disorders. Am J Surg. (2023) 226:485–91. doi: 10.1016/j.amjsurg.2023.06.003, PMID: 37330384

[32] Liu Y Yu X Wang Y Wu J Feng B Li M. The role of differentially expressed genes and immune cell infiltration in the progression of nonalcoholic steatohepatitis (NASH) to hepatocellular carcinoma (HCC): a new exploration based on bioinformatics analysis. Nucleosides Nucleotides Nucleic Acids. (2024) 43:1415–30. doi: 10.1080/15257770.2024.2310044, PMID: 38319987

[33] Zhu Q Li J Sun H Fan Z Hu J Chai S. O-GlcNAcylation of enolase 1 serves as a dual regulator of aerobic glycolysis and immune evasion in colorectal cancer. Proc Natl Acad Sci USA. (2024) 121:e2408354121. doi: 10.1073/pnas.2408354121, PMID: 39446384 PMC11536113

[34] Sun L Suo C Zhang T Shen S Gu X Qiu S. ENO1 promotes liver carcinogenesis through YAP1-dependent arachidonic acid metabolism. Nat Chem Biol. (2023) 19:1492–503. doi: 10.1038/s41589-023-01391-6, PMID: 37500770

[35] Zhang T Sun L Hao Y Suo C Shen S Wei H. ENO1 suppresses cancer cell ferroptosis by degrading the mRNA of iron regulatory protein 1. Nat Cancer. (2022) 3:75–89. doi: 10.1038/s43018-021-00299-1, PMID: 35121990

[36] Hu T Liu H Liang Z Wang F Zhou C Zheng X. Tumor-intrinsic CD47 signal regulates glycolysis and promotes colorectal cancer cell growth and metastasis. Theranostics. (2020) 10:4056–72. doi: 10.7150/thno.40860, PMID: 32226539 PMC7086360

[37] Chen LJ Li JYS Nguyen P He M Chen ZB Subramaniam S. Single-cell RNA sequencing unveils unique transcriptomic signatures of endothelial cells and role of ENO1 in response to disturbed flow. Proc Natl Acad Sci USA. (2024) 121:e2318904121. doi: 10.1073/pnas.2318904121, PMID: 38261622 PMC10835041

[38] Xu W Yang W Wu C Ma X Li H Zheng J. Enolase 1 correlated with cancer progression and immune-infiltrating in multiple cancer types: a pan-cancer analysis. Front Oncol. (2020) 10:593706. doi: 10.3389/fonc.2020.593706, PMID: 33643901 PMC7902799

[39] Dong G Adak S Spyropoulos G Zhang Q Feng C Yin L. Palmitoylation couples insulin hypersecretion with β cell failure in diabetes. Cell Metab. (2023) 35:332–344.e7. doi: 10.1016/j.cmet.2022.12.012, PMID: 36634673 PMC9908855

[40] Ehmsen S Pedersen RM Bang LL Asmussen A Kragh A Holm DK. BQ.1.1, XBB.1, and XBB.1.5 neutralization after bivalent mRNA COVID-19 booster in patients with cancer. Cancer Cell. (2023) 41:649–50. doi: 10.1016/j.ccell.2023.02.003, PMID: 36804967 PMC9910012

[41] Sun J Herazo-Maya JD Kaminski N Zhao H Warren JL. A Dirichlet process mixture model for clustering longitudinal gene expression data. Stat Med. (2017) 36:3495–506. doi: 10.1002/sim.7374, PMID: 28620908 PMC5583037

[42] Yang Z Wang G Luo N Tsang CK Huang L. Consensus clustering of gene expression profiles in peripheral blood of acute ischemic stroke patients. Front Neurol. (2022) 13:937501. doi: 10.3389/fneur.2022.937501, PMID: 35989931 PMC9388856

[43] Foessl I Haudum CW Vidakovic I Prassl R Franz J Mautner SI. miRNAs as regulators of the early local response to burn injuries. Int J Mol Sci. (2021) 22:9209. doi: 10.3390/ijms22179209, PMID: 34502118 PMC8430593

[44] Sun Q Hong Z Zhang C Wang L Han Z Ma D. Immune checkpoint therapy for solid tumours: clinical dilemmas and future trends. Signal Transduct Target Ther. (2023) 8:320. doi: 10.1038/s41392-023-01522-4, PMID: 37635168 PMC10460796

