# Supplementary material for: Transcriptomic profiling of burn patients reveals key lactylation-related genes and their molecular mechanisms
Source: Front Med (Lausanne). 2025 Jun 27;12:1554791. doi: 10.3389/fmed.2025.1554791 (PMC12245797; doi:10.3389/fmed.2025.1554791)
Supplement: Supplementary file 1 [file Data_Sheet_1.docx]

**Supplementary Materials**

### Transcriptomic Profiling of Burn Patients Reveals Key Lactylation-Related Genes and Their Molecular Mechanisms

**
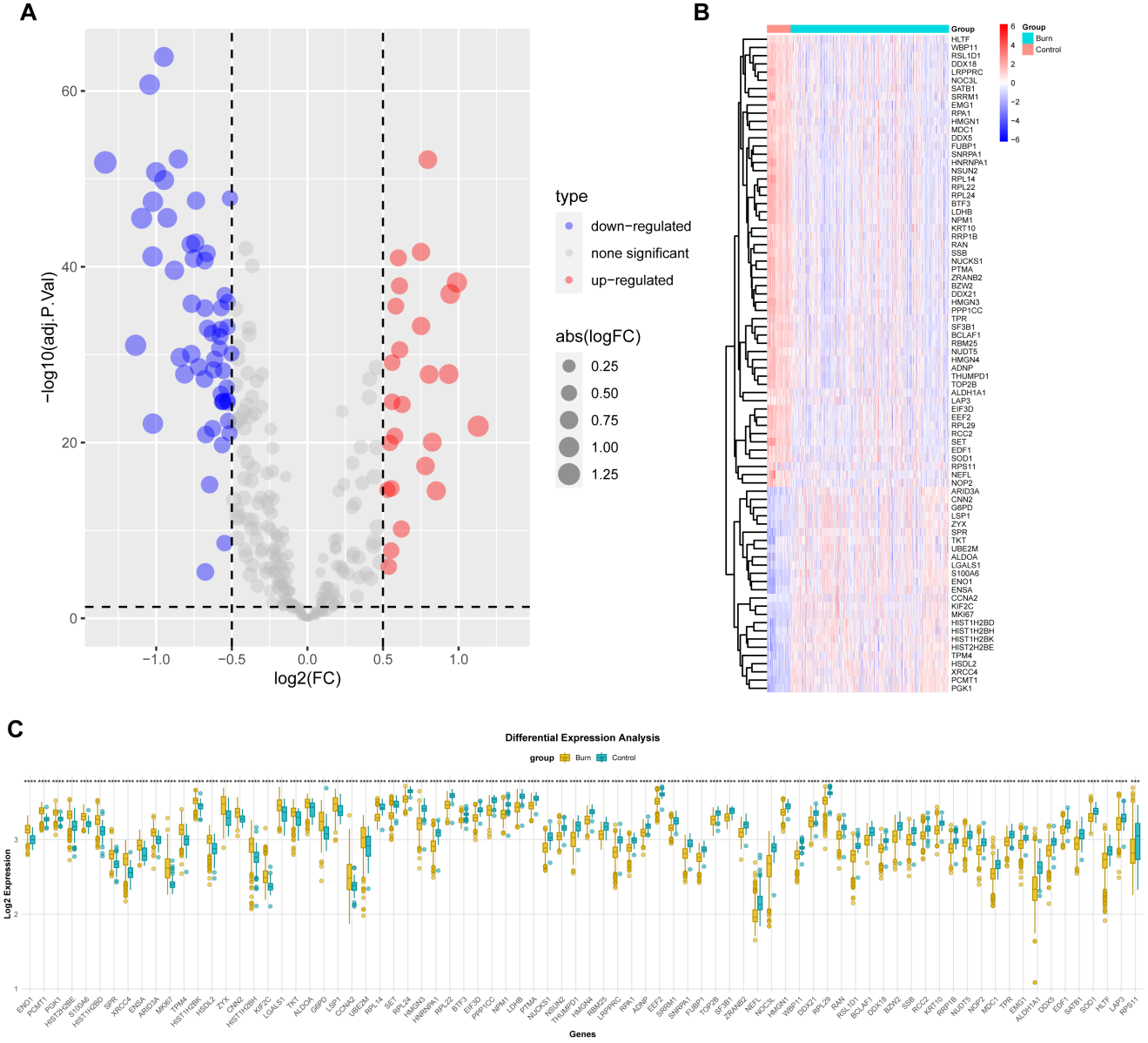
**

**FigureS1** **Expression Differences of Intersected Differential Genes** A. Volcano plot showing the expression differences of 80 intersected differential genes in patients and controls; B. Heatmap presenting the expression differences of 80 intersected differential genes in patients and controls; C. Box plot showing the expression differences of 80 intersected differential genes in patients and healthy controls.


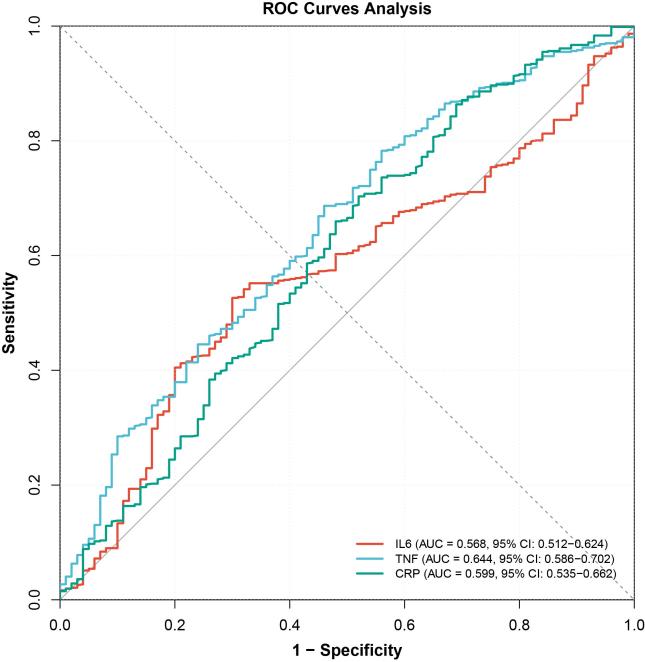


**FigureS2 ROC curves of the IL-6, TNF, and CRP for predicting burning occurrence.** The X-axis is "1-specificity", and the Y-axis is "sensitivity". Different genes have different sensitivities and specificities, and the area under the ROC curve (AUC) reflects the predictive ability of the gene for burning occurrence.


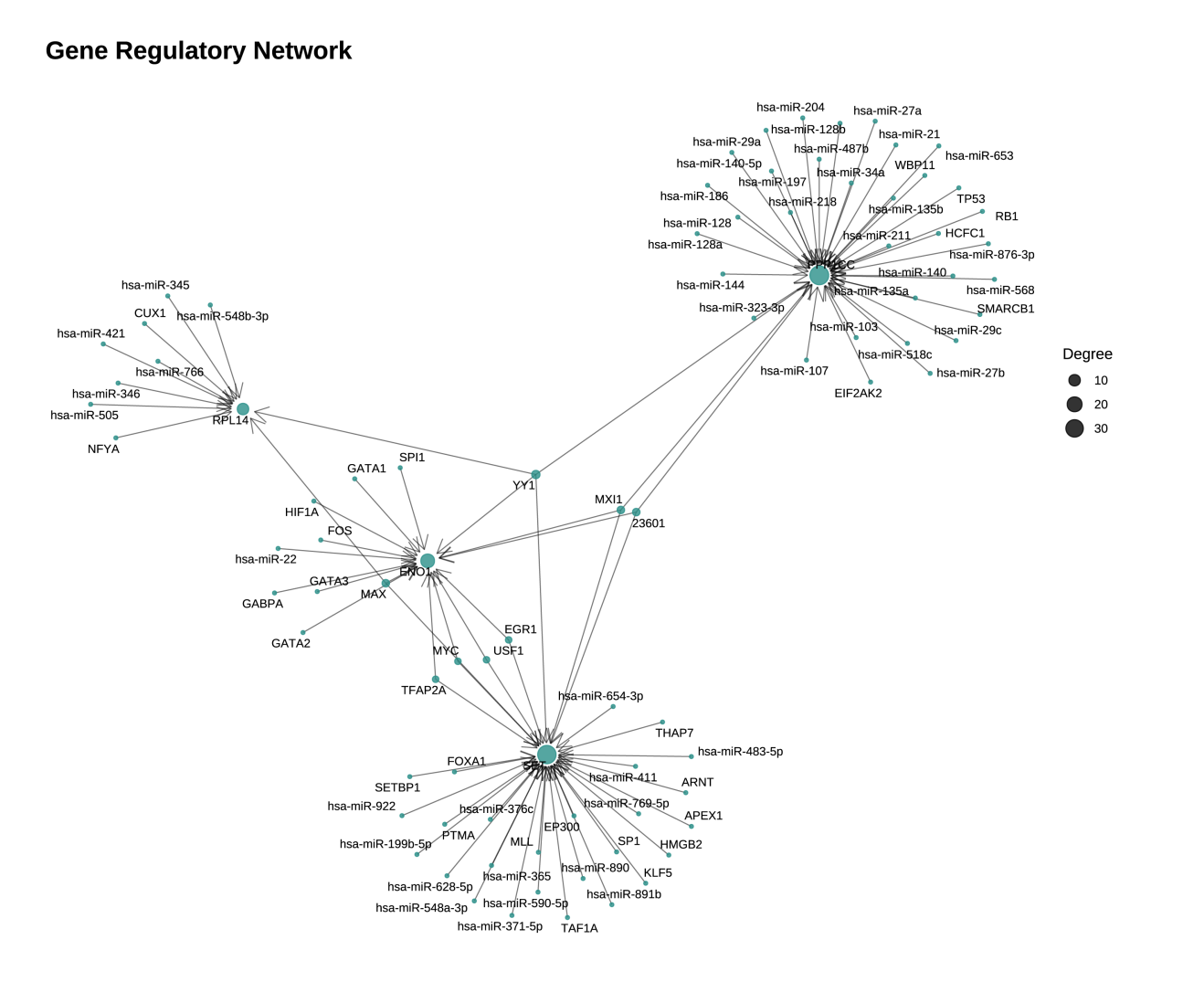


**FigureS3** Prediction of Upstream Regulatory FactorsNetwork graph showing the prediction of miRNAs and transcription factors upstream of the genes using the regnetwork database.
